# From neuroscience to evidence based psychological treatments – The promise and the challenge, ECNP March 2016, Nice, France

**DOI:** 10.1016/j.euroneuro.2017.10.036

**Published:** 2018-02

**Authors:** Guy M. Goodwin, Emily A. Holmes, Erik Andersson, Michael Browning, Andrew Jones, Johanna Lass-Hennemann, Kristoffer NT Månsson, Carolin Moessnang, Elske Salemink, Alvaro Sanchez, Linda van Zutphen, Renée M. Visser

**Affiliations:** a University Department of Psychiatry, University of Oxford and Oxford Health NHS Trust, Warneford Hospital, Oxford OX3 7JX, UK; b Department of Clinical Neuroscience, Karolinska Institutet, SE-171 77 Stockholm, Sweden; c Psychological Sciences, University of Liverpool, Bedford St South, Liverpool L697ZA, UK; d Division of Clinical Psychology and Psychotherapy, Department of Psychology, Saarland University, D- 66123 Saarbrucken, Germany; e Department of Psychology, Stockholm University, SE-106 91, Department of Clinical Neuroscience, Karolinska Institutet, SE-171 77 Stockholm, Sweden; f Central Institute of Mental Health, Medical Faculty Mannheim/Heidelberg University, J5, 68159 Mannheim, Germany; g Department of Psychology, University of Amsterdam, Nieuwe Achtergracht 129B, Amsterdam, the Netherlands; h Ghent University, Department of Experimental Clinical and Health Psychology, Henri Dunantlaan 2, B-9000 Ghent, Belgium; i Department of Clinical Psychological Science, Faculty of Psychology and Neuroscience, Universiteitssingel 40; 6229 ER, Maastricht University, Maastricht, the Netherlands; j Medical Research Council Cognition & Brain Sciences Unit, University of Cambridge, 15 Chaucer Road, Cambridge CB2 7EF, UK; k Department of Psychology, Uppsala University, SE-75105, Uppsala, Sweden

**Keywords:** Neuroscience, Cognitive Behaviour Therapy (CBT), Anxiety, Fear conditioning, Research Domain Criteria (RDoC)

## Abstract

This ECNP meeting was designed to build bridges between different constituencies of mental illness treatment researchers from a range of backgrounds with a specific focus on enhancing the development of novel, evidence based, psychological treatments. In particular we wished to explore the potential for basic neuroscience to support the development of more effective psychological treatments, just as this approach is starting to illuminate the actions of drugs. To fulfil this aim, a selection of clinical psychologists, psychiatrists and neuroscientists were invited to sit at the same table. The starting point of the meeting was the proposition that we know certain psychological treatments work, but we have only an approximate understanding of *why* they work. The first task in developing a coherent mental health science would therefore be to uncover the mechanisms (at all levels of analysis) of effective psychological treatments. Delineating these mechanisms, a task that will require input from both the clinic and the laboratory, will provide a key foundation for the rational optimisation of psychological treatments. As reviewed in this paper, the speakers at the meeting reviewed recent advances in the understanding of clinical and cognitive psychology, neuroscience, experimental psychopathology, and treatment delivery technology focussed primarily on anxiety disorders and depression. We started by asking three rhetorical questions: What has psychology done for treatment? What has technology done for psychology? What has neuroscience done for psychology? We then addressed how research in five broad research areas could inform the future development of better treatments: Attention, Conditioning, Compulsions and addiction, Emotional Memory, and Reward and emotional bias. Research in all these areas (and more) can be harnessed to neuroscience since psychological therapies are a *learning process* with a biological basis in the brain. Because current treatment approaches are not fully satisfactory, there is an imperative to understand why not. And when psychological therapies do work we need to understand why this is the case, and how we can improve them. We may be able to improve accessibility to treatment without understanding mechanisms. But for treatment innovation and improvement, mechanistic insights may actually help. Applying neuroscience in this way will become an additional mission for ECNP.

## 1 Introduction

The burdens and costs of mental illness for individuals and for society are enormous (for a review see Wittchen et al., 2011). The impact of these illnesses is not reflected in the level of resources directed towards the development of treatments for mental illnesses. This is because there is perceived to be a poor scientific understanding of the basis of mental illness and its treatments (Nutt and Goodwin, 2011). One factor that prevents greater traction is the relative fragmentation of the field into conceptual silos that focus narrowly on one approach to treatment development, using a pharmacological, psychological or social framework. This has resulted in parallel but separate efforts to develop treatments, often narrowly based on only one level of analysis, which do not leverage the advances made in other fields or draw on the potentials for synergies across fields.

Table 1 illustrates where the gaps in knowledge for drug and psychological treatment are most obvious at different levels of potential understanding. A biochemical level of analysis would apply to effects on neuronal receptors or neurotransmitters; it might prove target engagement for drugs as in radiotracer studies of receptor binding or monoamine turnover, possible in principle using positron emission tomography in man. Alternatively a genetic or other molecular marker might be established simply by pragmatic association studies. A systems target could reflect behavioural or neuroimaging measures apparently related to mechanisms mediating treatment efficacy. Cognitive theory is obviously strongly invoked in psychological treatments. Finally clinical features of individual patients may predict treatment outcome. All or any of these levels of analysis may contribute to treatment innovation and personalization with drugs or psychotherapy. At present the examples (shown as + for either treatment modality) are not numerous and in some boxes are completely absent (-). However, presented in this way the common ground for the traditionally separate drug and psychotherapy approaches appears obvious and could increasingly be nourished by advances in neuroscience.

**Table 1:** The gaps in understanding of how drug and psychotherapy may work. +++ indicates the domains where treatment innovation started. + indicates the domain where mechanistic studies are beginning and – indicates where contributions are yet to be made, but may be possible.

Drug	GAP	Psychotherapy
+++	Biochemical/Molecular	–
+	Neuronal system or functional domain	+
–	Cognitive theory	+++
+++	Clinical features	+++

This ECNP meeting was held in March 2016. It represented only one day together, but it was designed to build bridges between different mental illness treatment researchers from a range of backgrounds with a specific focus on enhancing the development of novel, evidence based, psychological treatments. In particular we wished to explore the potential for basic neuroscience to support the development of more effective psychological treatments (Holmes et al., 2014), just as this approach is starting to illuminate the actions of drugs. To fulfil this aim, a selection of clinical psychologists, psychiatrists and neuroscientists were invited to sit at the same table. Approximately 50% of attendees at the meeting reported that they combined clinical and research work.

The starting point of the meeting was the proposition that we know certain psychological treatments work, but we have only an approximate understanding of *why* they work. The first task in developing a coherent mental health science would therefore be to uncover the mechanisms (at all levels of analysis) of effective psychological treatments. Delineating these mechanisms, a task that will require input from both the clinic and the laboratory, will provide a key foundation for the rational optimisation of psychological treatments. As reviewed in this paper, the speakers at the meeting reviewed recent advances in the understanding of clinical and cognitive psychology, neuroscience, experimental psychopathology, and treatment delivery technology. We started by asking three rhetorical questions, the responses to which are summarized in the next paragraphs. We then addressed how research in four broad research areas could inform the development of better treatments.

We make no claim for this having been a comprehensive meeting. Its primary focus was anxiety and mood disorders and it was structured to promote discussion among all the attendees*, rather than didactically to cover the whole field*. Nevertheless, the content was of great contemporary interest and tended to confirm that we seem to be at a watershed in harnessing neuroscience for psychological treatment research, which represents a new mission for ECNP.

### 1.1 What has psychology done for treatment?

David Clark (University of Oxford) presented the decades of enormous progress made in the development of therapist-delivered treatments such as cognitive behavioural therapy (CBT) for a range of psychiatric disorders. Typically tailored for a given disorder, they have become more evidence-based, supported by RCTs with low drop-out rates and substantial effect sizes. The strategy clinical researchers use to develop and translate a promising cognitive theory into a new cognitive treatment was described (Clark, 2004): 1) identify core psychological abnormalities by means of careful clinical interviews and cognitive paradigms, 2) develop a theory of cognitive / behavioural processes that maintain the disorder, 3) test the theory of the maintaining factors with experimental psychopathology studies, 4) develop a cognitive/ behavioural treatment which targets the maintaining factors, and 5) test the efficacy and effectiveness of the treatment in a randomized controlled trial.

Close attention to phenomenology has identified apparently specific cognitive processes, such as negative beliefs and mental images, or problematic attention or memory processes, which maintain a given disorder, for example: Panic disorder (Clark, 1986); Social Phobia (Clark, 2001); Posttraumatic stress disorder (PTSD) (Ehlers and Clark, 2000); Obsessive compulsive disorder (OCD) (van Oppen and Arntz, 1994); and Generalized anxiety disorder (GAD) (Wells, 1999). These cognitive processes provide the conceptual target for treatment. For example, patients are trained to drop their safety-behaviours (i.e., internal mental or behavioural operations) in which patients engage to prevent imagined catastrophes from occurring (OCD; Deacon and Maack, 2008; PTSD: Dunmore et al., 1999; Social phobia: Kim, 2005; Wells et al., 1995; Salkovskis et al., 1999; Salkovskis et al., 2000; GAD: Woody and Rachman, 1994).

The resulting ‘cognitive models’ provide the clinical heuristic the therapist uses to formulate the case and discuss with the patient; they are not cognitive neuroscience models per se. They predict effects on the symptoms and can be tested pragmatically in research experiments manipulating the cognitive processes and the factors maintaining the disorder. When successful, versions of these experiments can be incorporated in the treatment protocol as so-called “behavioural experiments”. In CBT practice the term ‘behavioural experiment’ refers to an exercise whereby the patient tests out their predictions in reality with the aim to challenge their maladaptive beliefs; in neuroscience the same term refers to a research experiment conducted at a behavioural level rather than using neuroimaging or other direct measures of brain function. CBT behavioural experiments can afford an experiential proof to patients of the causal relations between a particular behaviour and a particular symptom complex e.g. in social phobia (Harvey et al., 2000).

Decomposition studies in which a full treatment is compared to a full treatment minus a certain procedure can in principle be used to simplify an overall recommended package of psychological treatment techniques, an approach in which Clark's group has been world leading. However, there is still considerable work to be done to create more focussed and briefer treatments. A particular difficulty is the inadequate power of most feasible therapist-led studies to detect small differences between treatments. In addition, improving or refining a CBT manual still assumes that it is a better average effect that is required rather than a more specific and personalized effect in the individual patient. That some patients do not respond to versions of cognitive treatment programs, represents a challenge to the adequacy of the explanatory model on which the treatment is based.

This approach of evidence-based CBT, with each protocol tailored for a specific disorder (e.g. panic disorder and social phobia), traditionally requires a high level of therapist competence; its availability inevitably limits access to treatment. There may be ways in which developing mechanism-oriented psychotherapy informed by neuroscience can overcome this limitation.

The challenge for any psychological treatment is to make it accessible on a large scale. The programme, Improving Access to Psychological treatments (IAPT) for CBT, has provided an important example in the UK (Clark, 2011). Lobbying and political initiatives were necessary to fund the dissemination of treatment studies into practice. It provides a remarkable example of implementation when clinicians themselves are sometimes sceptical about the generalizability of RCTs. The IAPT programme delivers evidence based individual therapy for anxiety and depressive disorders using traditional face-to-face CBT on a large scale. It has required training large numbers of IAPT-therapists to deliver specific protocols, without usually having the wider mental health professional background of a clinical psychologist, psychiatrist or mental health nurse. IAPT further provides systematically collected evidence of efficacy. The impact of this project on hard economic outcomes like employment remain to be further examined.

Looking forwards, if mass implementation of treatments can also be achieved via internet-based delivery rather than face-to-face, the current costs of treating only 15% of the clinical population could give access to all those who have the internet available. The challenge of using new technology was addressed as our second rhetorical question.

### 1.2 What has technology done for treatment?

The development of internet-based cognitive-behavior therapy (iCBT) was described by Christian Rück (Karolinska Institutet). The main focus of this section was What has the internet done for CBT, and scope of other technology was beyond the current discussion. His thought provoking metaphor was that the aim of iCBT is to *serve lobster at McDonalds*. There have already been over 150 randomized trials of iCBT. The patient does not attend a clinic, but instead logs on to a secure website and works with written self-help materials and homework assignments, which are closely monitored by a clinician. Thus, iCBT usually involves a therapist who provides support and feedback on each homework assignment. The main benefit of iCBT is a highly structured content and delivery; this standardization minimizes the risk of ‘therapist drift’ (Waller, 2009). Data fidelity is also better than in traditional trials because the primary outcome data can be entered directly into the research database rather than via more traditional paper-based methodology and adherence to the required therapy exercises can also be monitored precisely.

The internet psychiatry unit in Sweden (www.internetpsykiatri.se) forms part of regular health care in Stockholm for panic disorder, depression, social anxiety disorder and irritable bowel syndrome. It was founded in 2007 and has since treated over 4000 patients with iCBT. The patients first complete an online screening and, after that, see a clinician within 21 days. If suitable, the patient can start treatment within 7 days. Published effectiveness data show large within-group effect sizes (Andersson et al., 2015, El Alaoui et al., 2013, El Alaoui et al., 2015, Hedman et al., 2014). Another comparable example is the Mindspot clinic in Australia (www.mindspot.org.au), which treats about 15,000 patients per year. Thus, iCBT can remove many of the usual geographical and practical barriers that make access to CBT difficult for a large and dispersed population, but it also makes it easier for researchers to do large scale trials within a short timeframe (Andersson and Titov, 2014). While other countries are following this development, about 80% of all the RCTs on iCBT are currently from either Sweden or Australia which have large geographical areas of notably low population density (Arnberg et al., 2014). Internet treatment can facilitate research about the underlying mechanism of treatment or combination treatments. As examples, a dismantling trial by Ljótsson et al. (Ljotsson et al., 2014) randomized 309 patients with irritable bowel syndrome to receive either systematic exposure + mindfulness training vs. mindfulness training only. In another example, 128 OCD patients were randomized to either iCBT + D-cycloserine vs. iCBT + placebo (Andersson et al., 2015).

A further internet-based innovation that benefits mental health science is to refine how to find biomarkers of treatment response using data generated in the internet treatment process. A precision-based medicine approach can use “machine learning” on data sets to match patient with treatment for depression (Chekroud et al., 2016). As proof of principle on a smaller scale, fMRI data and machine learning techniques predicted long-term outcome of iCBT for social anxiety disorder (Mansson et al., 2015). Although larger replication trials are needed, preliminary results from studies using machine learning techniques on such data are promising. Given that the price of genome sequencing for the individual patient is falling steadily and dramatically, genetic stratification is also an imminent possibility. Combined with the large sample sizes possible in iCBT-trials, the potential to find biomarkers of treatment response is now obvious. However, the initial trials have not yet found significant genome-wide candidates (Coleman et al., 2016), which may mean larger sample sizes are required (or, of course, that underlying hypotheses need to be questioned). CBT is just one of many treatments that can be delivered by the internet and new develops are being tested though not as yet with clear success e.g. cognitive bias modification approaches (Boettcher et al., 2013). The scalability of internet studies may particularly facilitate the study of combination treatments (Williams et al., 2013).

Last but not least, the continuous development of virtual reality (VR) is a promising technological advance in the mental health field. VR has the potential to simulate specific real-life situations in which (exposure) therapy would be difficult (e.g., standing on the edge of a cliff to treat fear of heights; scenes of war to treat post-traumatic stress disorder). VR-products have been used in treatment studies for more than a decade but their full potential is yet to be realized (McCann et al., 2014).

If technology allows us to provide patients with effective, standardized and transparent treatments without any unnecessary waiting-time, key issues become acceptability and efficacy. Two recent trials in the UK did not find incremental effects of iCBT compared to treatment as usual (Gilbody et al., 2015, Phillips et al., 2014); suboptimal therapist support and high participant attrition was notable. Thus, iCBT may normally need facilitation by therapists and in any case, may not be right for everyone. Technology may facilitate the rapid conduct of better quality RCTs and potentially further enhance our understanding of efficacy and the testing of novel cognitive hypotheses. It is very likely to play a critical role in the translation of new ideas into practice.

### 1.3 What has neuroscience done for treatment?

Jonathon Rosier (UCL, London) explained that the last two decades have witnessed technical, computational and statistical developments in neuroscience, from subcellular mechanisms in the fruit fly nervous system to large-scale brain networks in humans. This knowledge has inspired new theories and shapes current views in psychology. But has this knowledge changed how mental health practitioners diagnose and treat mental disorders at all? To Roiser it seems self-evident that (ab)normal brain functioning has the potential to provide mechanistic insights into the etiology of mental disorders and the effectiveness of certain treatment. These insights could in turn inspire treatment development and optimisation. Yet, the answer to our third rhetorical question – what has neuroscience done for us? - is still “*not much*”.

The first obstacle may be the way we currently think about the diagnosis and etiology of mental disorders. A typical exam question for psychology undergraduates is “what causes depression?”. In their answers students may point to psychosocial causes, such as early mistreatment, thinking styles, or current life stress. Additionally, they may highlight biological causes involving genetic predisposition, effects of drugs, stress hormones etc. While all these factors have indeed been associated with Major Depressive Disorder (MDD), none of these factors shows a direct causal relationship with symptomatology across all depressed individuals. The diagnosis of MDD, is purely symptomatic. Asking “what causes a mental disorder?” is like asking “what causes a cough?”. This latter ‘syndrome’ has many potential causes, ranging from irritants, viruses, bacteria, tic disorders, asthma or even lung cancer. While these different causes may lead to the same symptom, they require radically different treatments. If we want to use neuroscience to gain insights into the etiology of mental disorders, we should start by recognizing as unlikely, that there will be a single responsible mechanism for descriptive diagnoses like depression or anxiety, and it is therefore unlikely that much can be revealed by a simple diagnostic test like a brain scan (Roiser, 2015).

The second point is, not only do we need to change the way we think about the *relation between* causes and symptoms, we also need to change the way we think about causes *per se*. In psychology, a distinction is often made between “biological *versus* psychosocial” factors that contribute to syndrome development. This is a false dichotomy, given that all behavior, including subjective experiences, are associated with brain activity. This brain activity is shaped by both genes and environment. For example, social factors (such as parenting style and early-life stress) have been shown to modify the expression of genes that influence brain structure and function. The science of epigenetics is still in its infancy, but such epigentic effects may influence the subsequent sensitivity of an individual to stressful stimuli (Weaver et al., 2004). In other words, our emotions, physiological response, action tendencies, and social behaviors are driven by - and at the same time shape – neurophysiological processes. Instead of “biological *versus* psychosocial”, a more sensible distinction would be to describe factors in terms of “proximal *and* distal” causes (Roiser, 2015). Proximal causes are *directly* related to the mechanisms driving symptoms (e.g., various forms of stress, drug effects or sudden loss), and are useful targets for *treatment*. Distal causes are *indirectly* related to the mechanisms driving symptoms (e.g., heritability, thinking styles, and early life experience), but may be useful targets for *prevention*. Importantly, both proximal and distal causes drive symptoms via modification of brain functioning.

Placing the brain at the center of psychological theories has many potential advantages. The NIMH's Research Domains Criteria (RDoC) has adopted a dimensional approach to understanding abnormal functioning; it moves away from describing disorders purely based on symptomatology (with its arbitrary boundaries), towards a clustering based on neurobiological functioning (Insel, 2013). Such a shift is controversial. Conventional diagnosis is based on the DSM methodology (American Psychiatric Association, 2013). It creates provisional categories that must be immediately useful in practice. For example, the MDD diagnosis is intended to identify patients for whom defined treatments are available. The relevant drug and psychological treatments have been tested in RCTs that included patients who cross this diagnostic threshold. A dimensional approach cannot currently serve the same purpose. Over time, perhaps, it could generate a diagnostic formulation that more closely maps onto neurobiological functioning, is putatively more ‘tangible’ and could eventually grant mental disorders the same status as (other) physical illnesses. It could eventually improve treatment selection and might additionally reduce stigma associated with mental disorders.

Arguably, one of the most exciting applications of neuroscience would be its guidance in individual treatment selection. Recent advances in the field of depression research suggest that such an application may not be entirely beyond reach. Experimental research has shown that depression is marked by disruptions in basic cognitive processing, including ‘cold’ cognitive deficits (e.g., reduced working memory capacity) and ‘hot’ cognitive deficits (e.g., impaired reward processing; altered responses to emotional faces) (Disner et al., 2011, Leppänen, 2006, Murphy et al., 1999, Rock et al., 2014), with the latter showing early improvement after treatment (Harmer et al., 2009a). Neuroimaging research has demonstrated robust depression-related anomalies in the subgenual anterior cingulate cortex (sgACC) across a range of tasks that tax ‘hot’ cognitive abilities (Ansell et al., 2012, Drevets, 2001, Drevets et al., 1997, Grimm et al., 2009, Harrison et al., 2009, Mayberg et al., 1999, O'Nions et al., 2011). Yet, substantial variability in neural responses across individuals has precluded the use of such scans for diagnostic purposes, fitting with the notion that depression is mechanistically heterogeneous (Roiser, 2015). Rather than viewing this heterogeneity as a problem, is has recently been argued that this variability is actually informative when systematically reviewed, inspiring new neuropsychological models of depression (Brooks and Stein, 2015, DeRubeis et al., 2008, Disner et al., 2011, Franklin et al., 2016, Roiser et al., 2012) and indicating that different treatments may work for different syndrome subtypes (McGrath et al., 2013, Roiser et al., 2012). For example, it seems that psychological treatments may be most effective in individuals with relatively normal baseline sgACC activity, while pharmacological treatments are more effective in individuals with abnormal sgACC baseline activity (Roiser et al., 2012). Systematically evaluating individual differences in neural activity patterns and treatment response could eventually allow us to move away from ‘one-size-fits-all’ approaches and towards patient-tailored treatments.

The subsequent sessions explored how the growing neuroscience and experimental psychopathology base in five research areas can currently inform the development of better treatments.

## 2 Attention

### 2.1 Gaia Scerif (University of Oxford) and Elske Salemink (University of Amsterdam)

Current models of attention (Corbetta and Shulman, 2002) highlight 1) the double influence of bottom-up stimuli salience and top-down processes in attentional functioning, and 2) how such attentional processes influence memory, learning and action. Thus, shifting of attention may be an important factor in determining how we learn, feel and behave in everyday situations. Critically, attention is disordered in a range of psychopathological conditions. These include anxiety, depression, ADHD, autism and schizophrenia. The focus of this session was on the interplay between attention, learning and interpretation processes (Gaia Scerif) and the implications of these findings for the development of novel clinical applications (Elske Salemink) primarily in the context of adolescent social anxiety.

In child development, learning processes and the factors that modulate their interplay are of particular interest (Amso and Scerif, 2015). Thus, attention patterns during learning influence subsequent memory performance, and memory encoding processes influence subsequent attention deployment in the presence of social distractors. These mechanisms may be essential to understand how social anxiety can emerge during development because of failures of attentional control (Francois et al., 2016). Cognitive-behavioral models of anxiety (Mathews and Mackintosh, 1998) have proposed that individuals with social anxiety preferentially allocate their attention to potential sources of threat. An overestimated interpretation of social threat in the environment will then generate avoidance behaviors involved in the maintenance of anxiety (Foa et al., 1986). Experimental evidence supports this proposal (Van Bockstaele et al., 2014). Attention bias modification (ABM) procedures have been developed to change attentional biases ‘bottom up’ by direct retraining of habits of thought, rather than ‘top down’ through verbal instructions and explicit challenge of dysfunctional thoughts, as in CBT (Baert et al., 2011). ABM is certainly associated with neural changes in lateral prefrontal cortex (Browning et al., 2010). Most of current ABM research has employed a variant of the visual dot-probe task (Amir et al., 2008); individuals are trained to orient attention away from negative information, according to contingencies between emotional stimuli (e.g., negative vs. neutral facial expressions) and subsequent probe detections (e.g., probe appearing in the location of the neutral expression). Initial results in adults with clinical social anxiety (Amir et al., 2008, Schmidt et al., 2009) were promising enough to suggest ABM as a clinical tool to change pathogenic mechanisms directly. This has not proved straightforward with mixed findings to date and debate about their significance (Cristea et al., 2015), but further research development is warranted.

The change in attentional bias achieved by the intervention (not always considered in various studies) may be essential for any ABM procedure to be effective in reducing social anxiety symptoms. If there is no abnormal bias to begin with, ABM is not likely to reduce anxiety symptoms and this is supported by a recent re-analysis of the Cristea et al. meta-analysis (Grafton et al., 2017). As a route to ‘precision psychology’, the specific populations that might benefit – or not – from these interventions and the number of sessions required to achieve a sustained change in pre-existing bias should be determined in future studies.

Improvement of current ABM approaches, and indeed other psychological treatment techniques that can harness attentional processes, is likely to involve targeting intentional top-down regulation of attentional functioning as well as simply training bottom-up salience contingencies (e.g., dot-probe ABM) (Sanchez et al., 2016). In addition, computerised training involves a large number of potentially tedious and repetitive sessions. We will need to enhance patient motivational to improve treatment adherence and ensure sustained benefits. Novel treatments will need to be compared with standard CBT (Blankers et al., 2016), as well as testing the combination of bias modification procedures with other therapies whether psychological or pharmacological (e.g., Browning et al., 2011; Williams et al., 2013).

## 3 Conditioning

### 3.1 Bram Vervliet (University of Leuven) and Andreas Olsson (Karolinska Institutet)

Bram Vervliet argued strongly that all psychotherapy can be viewed as a learning process with a biological basis in the brain. The challenge to psychotherapy of all descriptions is first to produce a desired change in behaviours, thoughts or feelings and, second, sustain the change. Fear extinction should be regarded as a particularly interesting translational model of behavior change. It is exemplary because fear extinction is easy to learn, but difficult to remember.

Exposure to fear provoking stimuli forms the basis of many effective therapies for treating anxiety disorders. The research evidence supports an inhibitory learning model for extinction, but this model has, hitherto, had little direct impact in clinical practice. However, substantial numbers of patients fail to benefit or relapse after treatment. Treatment failure may be related to measured deficits in the brain mechanisms that underlie exposure therapy. If these processes can be targeted, it would improve therapy efficacy. This may be achieved by more formal application of the ‘inhibitory learning’ model to optimize the impact of exposure in anxious patients. This approach can be distinguished from ‘fear habituation’ or ‘belief disconfirmation’ strategies common within standard CBT. Exposure optimization, based on this approach, offers preliminary evidence that model based enhancement of psychotherapy may be feasible and desirable (Craske et al., 2014).

Improved understanding of such a model will depend on advances in neuroscience. Animal experimentation will also be fundamental to the elaboration of learning models of psychotherapy. There is a long history in pharmacological research of screening novel drug compounds in ‘animal models’ of psychopathology. Criteria have been developed to estimate any model's external validity using concepts such as face, predictive and construct validity; in other words, how closely can the model be said to have properties relevant to, or identical with, the target disorder. Behavioural models can also be used to study the *psychological* processes underlying disordered behaviour. The same criteria employed in pharmacology are relevant to this research. Furthermore, diagnostic validity may be an added criterion of validity in this application. Models of anxiety and depression can be shown to possess face, diagnostic and construct validity. However, direct tests of predictive validity are usually absent and could provide important additional support (Vervliet and Raes, 2013).

If fear extinction is a key model, progress will require studies of extinction of de novo conditioned fears in animals with the key translational step being its extension to confirm related mechanisms in the extinction of de novo conditioned fears in anxious human individuals. The locus of the neurobiology of extinction appears to lie in circuits linking dorsal anterior cingulate (dACC) / ventromedial prefrontal cortex (vmPFC) and the amygdala (Milad et al., 2009). Early findings with drugs that may enhance learning have been mixed, but the principle is clear. Pharmacological enhancement of psychotherapeutic processes may be possible alongside behavioural enhancement of the same psychotherapeutic processes. Biofeedback (e.g. real time fMRI monitoring of vmPFC involvement) may be a further elaboration. The expectation is improved treatment, especially for patients who do not respond to simpler interventions. The untapped potential and possibility for synergies makes this approach exciting.

Andreas Olsson reflected on how Research Domain Criteria (RDoC) refocus on a number of psychological constructs linked to behavioral dimensions with known neural circuitry. The domain framework recognizes five constructs: arousal/modulation, cognition, negative valence, positive valence, and social processes. Conditioning is a basic mechanism that crosses these psychological constructs, has clear behavioural dimensions and a known brain circuitry.

Traditional fear conditioning paradigms have not included an important social dimension that must be important in conditioning that takes place in a natural environment. Indeed, in rodents, transmission of social conditioning has been demonstrated between cage-mates (Bruchey et al., 2010) and even across generations (Askew and Field, 2008, Debiec and Sullivan, 2014). This data has parallels with the self-reported origins of phobias: 57–78% of people with phobias remembered direct conditioning, 17–42% remembered learning from *vicarious experiences* and 10–25% from verbal information only (Askew and Field, 2008). Indirect or vicarious experiences were also included in DSM-IV criterion for PTSD (American Psychiatric Association, 2013). Such social conditioning has been suggested to be a major pathway for intergenerational transmission of anxiety disorders (Eley et al., 2015, Ginsburg et al., 2015).

Thus, there is the potential to capitalize on social information to enhance models of the cause and treatment of psychiatric disorders, and arguably its resolution. Indeed, face-to-face psychological therapy is an inherently social process. Modeling-based exposure has long been proven effective in treatment of specific phobias (Bandura et al., 1967) and observational fear learning and extinction is beginning to be mapped in the brain (Olsson et al., 2015, Olsson and Phelps, 2007).

## 4 Compulsions and addiction

### 4.1 Matt Field (University of Liverpool) and Reinout Wiers (University of Amsterdam)

Contemporary theoretical models suggest addictive behaviours develop and are maintained through the interaction of two qualitatively distinct systems (Wiers et al., 2007, Wiers et al., 2013). These ‘dual-process’ models suggests an imbalance between an impulsive system which becomes sensitized following repeated drug-use, and a regulatory process which serves to moderate the impact of the impulsive drive.

The impulsive system is identified with the appetitive motivation to obtain and consume drugs of abuse. The strength of this system is determined through relative contributions of pharmacologically enhanced learning processes, including but not limited to; incentive salience, habit formation, and negative reinforcement. Behavioural manifestations of a sensitized impulsive system include cognitive biases, such as increased attention and approach for drug-related stimuli (Wiers et al., 2007). These behaviours can be readily investigated in the laboratory using computerised tasks. For example, approach biases can be measured by individuals pulling or pushing a joystick to approach or avoid a drug-related cue respectively, with faster reaction times when approaching compared to avoiding drug-related cues indicative of an approach bias. Attentional and approach biases have been demonstrated in clinical and non-clinical populations, and predict quantity and frequency of use across different drugs (Cousijn et al., 2011, Field et al., 2009, Sharbanee et al., 2013).

The regulatory processes that moderate the impact of impulsive reactions on behaviour are executive in nature, and include working memory and inhibitory control. Inhibitory control is thought to be particularly relevant to addiction: it is the ability to stop, change or delay a response that is no longer appropriate (Logan et al., 1984). As a higher order process, it overlaps substantially with broader concepts such as impulse regulation and self-control (Baumeister, 2014). Inhibitory control can be modelled in the laboratory using the Stop Signal or Go/No-Go tasks (Verbruggen and Logan, 2008). These tasks require individuals to inhibit a pre-potent motor response when presented with an environmental signal to inhibit. Impairments in inhibitory control as measured using these tasks are robust across different substances of abuse, with a recent meta-analysis demonstrating a small but consistent effect (Smith et al., 2014).

If the interaction between compulsion and control determines behaviour (Friese and Hofmann, 2009, Thush et al., 2008), weakening the impulsive processes or strengthening the control processes should lead to novel behavioural interventions for substance use (Friese et al., 2011). One approach to a psychological intervention to *weaken* the impulsive system, is via computerised Cognitive Bias Modification (CBM). Within CBM, Approach/Avoidance Training (AAT) is a behavioural paradigm in which individuals make a response to avoid substance-related cues. Proof-of-concept studies in the laboratory have led to reductions in approach behaviour and also concurrent reductions in subsequent alcohol consumption (Wiers et al., 2010), compared to a control condition in which alcohol-related cues were approached. Translation of these findings into the clinic has also led to clinically relevant outcomes, with alcoholics trained to avoid alcohol-related cues showing significant reductions in relapse rates up to one year following training (Eberl et al., 2013; ~10%; Wiers et al., 2011). However, null findings have also been reported in heavy drinking samples (Lindgren et al., 2015, Wiers et al., 2015) suggesting the need for further research (see also the Attention section above for debate concerning this field).

The alternative (or complementary) approach targets the other component of the dual process model, by seeking to *strengthen* the control or regulatory systems. Substance-using individuals exhibit impaired inhibitory control to drug-related cues (Jones and Field, 2015), creating high-risk situations in which individuals are more likely to consume substances or relapse (Jones et al., 2013). Inhibitory Control Training (ICT) prompts individuals to associate substance-related cues with inhibitory responses, with the objective of inhibition or ‘stopping’ of problem behaviour outside the laboratory (Verbruggen et al., 2014). Recent meta-analyses demonstrated a small but robust effect on appetitive behaviour change in the laboratory (Allom et al., 2015, Jones et al., 2016) and proof-of-concept ICT has led to reductions in alcohol consumption relative to control groups who do not inhibit to alcohol-related cues (Jones et al., 2013). An important challenge - translational research using repeated ICT in heavy drinkers - is ongoing (Jones et al., 2014, Van Deursen et al., 2013). We may be encouraged by observations from the parallel literature in obesity which have demonstrated weight loss and reduction of calorie intake following repeated online ICT (Lawrence et al., 2015, Veling et al., 2014).

The emergence of computerised CBM and ICT approaches for addiction presents benefits over traditional ‘face-to-face’ psychological treatments. They can be delivered at low cost via the internet or smartphones, so avoiding geographical barriers and the presence of a healthcare professional. However, underlying mechanism(s) and potential moderators are still under investigation, for example does ICT result in a *strengthened* control system or extinction of approach behaviour (Jones et al., 2016)? Furthermore, engagement with training is reliant on individual's motivation to change, and compliance without the presence of a healthcare professional is often poor (Wiers et al., 2015). As noticed above, the development of such novel mechanistically-based psychological treatment interventions may still require clinical expertise in treating the illness / symptom in question, in this case addiction.

## 5 Emotional memory

### 5.1 Tanja Michael (University of Saarland) & Merel Kindt (University of Amsterdam)

We are what we remember and human behavior is largely determined by learning and memory processes (Roberts, 2014). Thus, our learning experiences and therefore our memories shape our behaviour and our identity. The rapid acquisition of negative emotional memories is usually viewed as an adaptive response to ensure the survival of an individual in potentially harmful situations. However, this adaptive process is distorted in anxiety disorders, which are characterized by a strong persistence and generalization of fear to novel stimuli and contexts in the absence of actual danger (Kindt, 2014). The attempt to modify these maladaptive memories is the main aim of CBT for anxiety disorders. CBT is, on average, an effective treatment, however, about 50% of patients do not respond (Holmes et al., 2014) and return of fear and relapse after CBT is a common phenomenon in anxiety disorders (Craske et al., 2014).

While emotional memories are easy to acquire, they are strong and resistant to change: the session focussed on new insights on mechanisms and strategies to modify emotional memories in order to enhance treatment for anxiety disorders. One approach to strengthening re-learning in therapies is clearly through changes in the chemical environment of the brain - whether induced behaviourally or pharmacologically - and recently, several pharmacological agents have been proposed as boosters of exposure therapy for example cycloserine which has been tried in several disorders (for a review see Hofmann et al., 2014).

### 5.2 Cortisol as a potential pharmacological booster of exposure therapy

Cortisol is a steroid hormone, which is secreted in response to stress and has several influences on body and brain functioning. It has been shown to enhance the consolidation of newly acquired material and inhibit the retrieval of previously learned material (de Quervain et al., 2009). This characteristic of cortisol makes it a promising tool to enhance exposure therapy for anxiety disorders (Bentz et al., 2010) and observational findings showed a reduction in phobic fear after cortisol administration in patients with spider phobia und social phobia (Soravia et al., 2006). The first double-blind placebo controlled trial on the effects of cortisol administration on exposure therapy in height phobia showed a significantly greater reduction in fear of heights both at post-treatment and at follow-up (de Quervain et al., 2011). These findings were replicated in a placebo controlled trial with spider phobics (Soravia et al., 2014).

Cortisol levels are circadian, with high levels in the morning and low levels in the evening. This predicts better outcomes for exposure therapy conducted in the morning, compared with the evening, which was confirmed for fear of spiders (Lass-Hennemann and Michael, 2014). It provides an important proof of concept for therapy enhancement through naturally occurring hormones. Future research should focus on the effects of cortisol administration on exposure treatment for more complex anxiety disorders.

### 5.3 Cortisol, PTSD and intrusive memories

Cortisol has also been proposed as a treatment adjunct for PTSD patients, who suffer from intrusive memories of the traumatic event (de Quervain and Margraf, 2008). Intrusive memories reflect the uncontrolled and excessive retrieval of traumatic memories (Ehlers et al., 2004). Because of the retrieval-inhibiting effects of cortisol, continuous cortisol administration is hypothesized to reduce intrusive memories in PTSD. Michael presented two analog studies showing that acute cortisol administration was able to reduce perceptual priming for neutral stimuli in a traumatic context, a memory process which has been shown to underlie intrusive memories (Holz et al., 2014). A second analog study used the trauma-film-paradigm (an experimental tool for investigating intrusive memories in healthy participants; Bourne et al., 2013; Clark et al., 2016; James et al., 2015; James et al., 2016). Cortisol administration over 3 consecutive days did *not* inhibit intrusive memories to the traumatic film clip (Graebener et al., 2017). This is in line with a recent study in female patients with complex PTSD that also did not find a reduction in intrusive memories after repeated cortisol administration (Ludascher et al., 2015). While there is some evidence that cortisol administration directly after a traumatic event might prevent PTSD (Hauer et al., 2014), work is needed to clarify the exact circumstances under which cortisol may be beneficial in reducing the psychological impact of trauma, and on the core clinical feature of intrusive memories of trauma in particular.

### 5.4 Reconsolidation

As described above, extinction is an inhibitory learning process, in which a new memory trace is formed, which inhibits the ‘old’ fear-related memory trace. However, while the fear-memory is very stable and easily generalizes to another context, the new inhibitory memory trace is rather fragile and context dependent. If, after extinction/exposure therapy, the fear memory is still intact, it may resurface, leading to return of fear and to relapse (Craske et al., 2014). A promising alternative to modify fear memory is to target the original fear memory trace directly, by disrupting its reconsolidation. Reconsolidation occurs after retrieval of a previously stable memory has brought that memory into a transient labile state. In the few hours that it takes for a memory to return to its stable state (i.e., to ‘re-consolidate’) it is susceptible to change. Thus, interfering with the process of reconsolidation offers the advantage of directly targeting the original fear memory (Kindt et al., 2014).

Pharmacological disruption of the reconsolidation process was observed first in animal studies (Misanin et al., 1968, Nader et al., 2000, Przybyslawski and Sara, 1997). It was translated to fear conditioning in healthy volunteers (Kindt et al., 2009) by the administration of the beta-blocker propranolol (which inhibits noradrenaline-stimulated CREB phosphorylation in the brain, Jockers et al., 1998). Propranolol administered before or after memory activation reduced the conditioned fear response a day later, and prevented the return of fear (Kindt et al., 2009). The findings were replicated in several independent samples (e.g., Sevenster et al., 2013; Sevenster et al., 2014; Soeter and Kindt, 2010, Soeter and Kindt, 2011, Soeter and Kindt, 2012). Importantly, they found that the administration of propranolol after memory activation not only eliminated the fear response a day later, but also blocked reinstatement, rapid reacquisition, and spontaneous recovery of fear. There is one study to date which did not replicate the same pattern of results (Thome et al., 2016). Overall, the evidence argues clearly for the idea that propranolol can permanently modify the original fear memory trace.

Although fear conditioning studies are thought to be a good experimental model for pathological anxiety, it is not clear whether the disruption of memory reconsolidation for ‘just acquired’ fear-conditioned responses, readily transfers to pathological fear and anxiety, which are based on stronger and older fear memories. In a recent study of high spider anxious individuals, a very short exposure to a live-spider (2 min) followed by the intake of 40 mg propranolol was effective in reducing spider fear behaviour at posttreatment and at follow-up (Soeter and Kindt, 2015). However, there have also been discouraging results in three studies of PTSD patients who showed no reduction in PTSD symptoms after attempted pharmacological blockade of memory reconsolidation (Wood et al., 2015).

### 5.5 Different expressions of emotional memory

There may be a dissociation in sensitivity to the reconsolidation-propranolol procedure, between different read-outs of emotional memory. Laboratory findings have shown that propranolol solely affected the amygdala-dependent startle reflex, while leaving threat expectancies unaffected (Soeter and Kindt, 2010). Correspondingly, in high spider anxious individuals, the reduction in spider fearful behaviour after memory reactivation plus propranolol, was not accompanied by reduced subjective ratings of fear of spiders (Soeter and Kindt, 2015). A change in self-declared fear of spiders followed several month later. Cognitive models suppose dysfunctional cognitions and beliefs to be at the core of anxiety disorders; changing those beliefs would be a prerequisite for an effective anxiety treatment, which is incompatible with these findings.

The theme of the session was that a minor change in the brain's chemical environment can enhance the learning processes underlying the treatment of anxiety disorders. However, the direct translation of basic neuroscience research to clinical practice is painstaking. Small steps may be required to refine the optimal doses and timing of drug administration to bridge the gap between insights from basic neuroscience research and clinical practice. However, the potential for using pharmacological agents creatively to enhance learning or extinction procedures is both obvious but, as yet largely unexplored.

## 6 Reward and emotional bias

### 6.1 Catherine Harmer (University of Oxford) and Andreas Mayer-Lindenberg (University of Mannheim)

Aberrant reward and emotional processing are present in many psychiatric disorders. An important step towards the improvement of psychological treatment is to characterize disease-specific phenotypes of disturbed reward and emotional processing at various levels, including behavioural readouts and underlying neural circuitries. The aim is to identify specific and reliable markers as a surrogate of the disease and of induced therapeutic effects.

Negative affective bias has provided an obvious starting point for such studies in depression (Harmer and Cowen, 2013, Warren et al., 2015). At the behavioural level, emotional bias has been assessed in tasks probing the ability to recognize the emotional expression of happiness in facial stimuli, and the categorization and recall of self-relevant positive personality traits. In the acute depressive state, patients performed significantly worse than control subjects as a result of a negative processing bias (and impaired episodic memory) (Harmer et al., 2009b). In healthy volunteers, the administration of an antidepressant (e.g. the noradrenaline reuptake inhibitor reboxetine or the selective serotonin reuptake inhibitor citalopram) led to a significant increase in performance (Harmer et al., 2004), even as early as 3 h after an acute dosage (Harmer et al., 2009a). The same effect was seen in depressed patients treated with reboxetine (Harmer et al., 2009b). In contrast, the effect of antidepressants on self- or observer- rated mood can only be observed after a longer delay of several weeks.

A compelling explanation is that the mood-enhancing effects of pharmacological agents are mediated via their direct action on emotional processing. By reducing, or even abolishing, the bias towards negative emotional processing, patients may be enabled to experience their environment in a new, more positive way, which ultimately results in symptom relief (Browning et al., 2012, Harmer et al., 2009a). The validity of this neurocognitive model is supported by other findings. First, changes in emotional bias are predictive of treatment outcome (Shiroma et al., 2014, Tranter et al., 2009). Second, the changes in positive memory bias and subsequent mood enhancement are specific to drugs effective in treating depression; similar effects are not seen with a purely anxiolytic agent (e.g. diazepam; Murphy et al., 2008) or in placebo conditions. Third, the mediating effect of negative bias reduction on symptom relief was moderated by the degree of social support (Shiroma et al., 2014). Fourth, the early effects of pharmacological agents on emotional bias are associated with early changes in functional neural circuitries implicated in emotional processing, including amygdala, anterior cingulate cortex and medial frontal cortex (Warren et al., 2015). These early changes in emotional processing are the first plausible example of a biomarker to aid treatment selection. Thus, failure to show an early effect of an antidepressant may be a predictor of subsequent non-response in both clinical trials and ordinary practice.

Intriguingly, there is evidence that the same neurocognitive model holds for at least one psychological treatment. A single session of exposure based CBT in patients with panic disorder led to a marked reduction of emotional bias towards threat-related information on the post-treatment day. However, self-rated symptoms were unchanged at that stage. The early change in emotional bias predicted symptomatic outcome at a 4-week follow up (Reinecke et al., 2013). Similar to the work on attentional bias modification, a change in symptoms first and foremost requires a change in bias.

This neurocognitive approach, by offering a worked example of a psychopathological biomarker, offers other ways to enhance treatment response for depression. It predicts that behavioural activation therapy will strengthen, or even accelerate, the effects of bias modification by promoting positive experiences in the subjective phase recovery. More mechanistically, different treatments that rely on the same mechanism of early bias modification, e.g. CBT and pharmacological agents for panic disorder, need to be tested for synergistic (e.g. super-additive) or potential adverse (e.g. interfering) effects (Browning et al., 2011).

The neurocognitive model of emotional bias has its origins in the study of behaviour measures (i.e. negative affective bias) which are an extension of the relevant phenotype. A more radical alternative route is the in-depth characterization of the neural networks related to reward and emotion processing and their interplay with other neural systems. Functional neuroimaging can directly map these systems and therefore has a great potential to inform the development and evaluation of psychological treatment because they represent the primary locus for target engagement by any intervention. Biomarkers in the emotional and motivational domain are also suitable for translational research, because they are highly preserved across species. The back-translation into the animal domain offers the opportunity to inform the development and evaluation of novel treatments targeting the reward circuitry.

Numerous studies have shown the ubiquity of affective and motivational dysfunction across psychiatric conditions and their interaction with other cognitive systems. This calls for a trans-diagnostic and dimensional approach involving large samples sizes in order to obtain biologically plausible readouts (Buckholtz and Meyer-Lindenberg, 2012). A “battery” approach is promising if it allows for the reliable and time-efficient assessment of functional networks across domains within one single session (Braun et al., 2012, Cao et al., 2014, Loth et al., 2015, Plichta et al., 2012). The goal is the extraction of biomarkers from these highly complex data sets by means of multivariate analyses techniques (Frangou et al., 2016).

Emotion and reward processing are influenced by the immediate environment and well-known ‘environmental’ risk factors for mental illness have been identified in epidemiological studies. Thus, social status, ethnic minority status or urban upbringing (for a review see Meyer-Lindenberg and Tost, 2012) are reflected in activation changes in primary subcortical (e.g., amygdala) and cortical control areas (e.g., anterior cingulate cortex) of the reward and emotion processing systems (Akdeniz et al., 2014, Haddad et al., 2015, Lederbogen et al., 2011, Zink et al., 2008). In order to uncover the mechanisms underlying risk or resilience, a novel approach with high ecological validity has been put forward in recent research. The idea is to study individuals in their daily environment and assess their emotional and motivation states in dependence of environmental factors, using ambulatory data from smartphones (Ebner-Priemer et al., 2012). The aim is to relate these interactions to neural circuits, as successfully demonstrated in a recent study on real-life positive affect and neural reward processing (Heller et al., 2015), and to study these interactions in a developmental framework for obtaining systems-level markers of environmental risk (Tost et al., 2015).

The social environment is further constructed by dyadic interactions with social partners (including in the specialized setting of psychotherapy) (Horvath et al., 2011). These interactions can be studied in so-called hyperscanning scenarios, where the simultaneous measurement of social partners allows for the tracking of information flow between interacting brains (Bilek et al., 2015). This approach bears the opportunity to characterize the neural correlates of disturbed social interaction in psychiatric disorders, their relation to affective symptomatology, and their modulation by pharmacological or psychological treatment. Of special interest in this respect is the development of drugs, related to the neuropeptides oxytocin and arginine vasopressin, which influence pro-social behaviour and which have been shown to modulate neural circuitries for emotion and social cognition (Kirsch et al., 2005, Meyer-Lindenberg et al., 2009, Tost et al., 2010, Zink et al., 2010). According to the psychobiological therapy approach, the effects of established cognitive-behavioural protocols could be supported by pharmacological enhancement of central socio-affective processing (Meyer-Lindenberg et al., 2011).

## 7 Conclusions

It is not difficult to identify the need for improved psychological treatments. There was broad agreement that a major challenge in clinical practice is that treatment response is highly individually variable. Particular treatments are described as efficacious for particular patient populations but they do not help everyone, and it is potentially as important to understand why a treatment has failed as why it has worked. The responses to this unmet need for better patient-tailored treatments were several. However, the common themes were the improvement of average psychological treatment effects and/ or the personalization of treatments. More effective treatments may require refinement of current approaches or innovation to develop new ones. Personalization of treatments may be achieved by improved diagnosis or baseline measurement, so that the choice of approach will be optimal. Alternatively, measures of early effects on for example emotional bias or even neurobiology (target engagement) may allow early modification of a treatment to achieve greater efficacy more efficiently than by clinical trial and error. A finer grained appreciation of treatment mechanism may be an essential first step to identifying personally tailored treatments. This represents a universal call for biomarkers. Biomarkers are essentially measures of effect proximal to the site of action of a drug or psychological intervention. They are not necessarily ‘biological’ – and could be behavioural or cognitive for example - but they do reflect the experimental framework used to aid scientific traction.

### 7.1 Should we improve accessibility to treatment, without understanding mechanisms?

Behaviour therapy and CBT, with their roots in behavioural science, represent a fundamental advance over traditional, analytically derived psychotherapy. There is not yet a complete consensus that a more detailed understanding of the microenvironment of emotional learning is required for progress. CBT has been successfully adopted on a wide scale for the treatment of the anxiety disorders. It builds on models of psychopathology, which are pragmatic and usually unsupported by neurobiology. But they work. It is possible to increase access by creating online programmes with or without relatively inexpert therapy support.

The advances in information technology make trials easier to conduct with potentially better controlled comparison conditions. Average treatment effects may be further improved. In addition, machine learning may make online interventions more intelligent and better tailored to the individual patient. In the past, a particular challenge for psychological interventions has been the design of adequately controlled trials, the demand characteristics introduced by non-blinding and the poor scalability of effective treatments. There is clearly enormous scope for improvements in the quality and credibility of clinical trials and the provision of low-cost computerised interventions that are accessible to large populations just by adopting and extending online/mobile methodology. This can occur without invoking theories grounded in neuroscience and will apply to most pragmatic psychotherapies. Increased scale could lead to improved measurement of relevant social outcomes like employment and economic success.

### 7.2 Do we need to understand existing treatment models and hence their mechanisms?

For treatment innovation and improvement mechanistic insights actually help. It was widely expressed that a multidisciplinary, evidence based approach provides the ideal platform from which to develop novel psychological treatments and that recent advances in psychology, neuroscience and technology make this the ideal time to push forward in new directions – and to do so with synergies across disciplines.

Fear conditioning provides one key mechanism linking basic animal research and studies in healthy volunteers. The basic science of fear conditioning has been galvanized by fascinating new methods, based on optogenetics in particular, that allow the dissection of individual pathways in unconstrained animals. Very chemically specific measures can be made and a much more sophisticated account of animal models of fear-related behaviour is increasingly feasible. Advances of this kind promise a realization of the wish to understand the chemical environment in which learning occurs. It will also allow a more profound understanding of how interventions sensitize the reward system or reduce pain. Equivalent, necessarily invasive studies in man are not possible. However, the potential to study novel anxiolytic drugs / behavioural treatments in essentially similar models that translate across animal and human models is likely to be greatly strengthened. In this way neuroscience may directly facilitate the development of novel treatments. The potential to use new or existing drugs to facilitate the mechanisms underlying psychological treatments like exposure/de-sensitization – i.e. combination treatments - is also clearly possible. Both pharmacological and psychological therapies have a biological basis in the brain and their synergies remain to be better understood.

Innovation can proceed from systems level behavioural science as well. The identification of the core psychological systems and processes which are impaired through addiction has led to the development of novel behavioural interventions, via a translational research pathway. Whilst relatively novel, some of these approaches are beginning to show promise when translated to the clinic and will need testing in comparison with CBT and pharmacotherapy.

### 7.3 Are biomarkers going to be possible?

The early change in emotional processing produced by antidepressants is the first plausible example of a biomarker to aid treatment selection in psychiatry. Generalization of this particular approach to psychotherapy may depend on how far early target engagement and implicit changes in emotional processing provide a common pathway for drug and psychological therapy effects. Measures of effects on emotional bias may be highly relevant to the forms of psychotherapy described for attention and fear conditioning in previous sections. They may be less relevant to top down, reflective processes believed to be involved in CBT. However, the way in which patients engage in computerized therapies may provide data that lends itself to analysis of processes in therapy that can predict outcomes. The cumulative evidence from psychotherapy and neuroscience suggests a central role of reward and positive affect for the success of psychological treatment. This calls for a stronger emphasis on methods which increase the motivation of patients, such as promoting therapies in a resilience framework, empowering patients by playing back study data, by providing regular feedback and so forth.

### 7.4 Limitations

Clinical psychology has, within the wider mental health arena, traditionally been seen as providing treatments for anxiety disorders, substance use and more recently depression; most, perhaps all of the discussions at this meeting were confined to these clinical areas. The role of clinical psychology in relation to schizophrenia and bipolar disorder was not discussed. There is a clear need for future meetings on psychological treatments and neuroscience and for them to also cover such additional, important areas of mental disorders.

Clearly, we do not yet know whether neuroscience research can profoundly assist psychotherapy research, without first transforming our diagnostic practice. Moreover, we do not know if the tools of human neuroscience are sufficiently precise to effect that transformation. Advances are likely to require new frameworks - such as a focus on core clinical features rather than full diagnoses - and to combine a focus on process and mechanisms within a theoretical framework underpinning symptom change. The endeavour may take time to yield fruit – and research advance in this pursuit should not be at the expense of research using other methods to improve therapies. But nothing is lost by promoting links between brain-based and mind-based theory; we already know that ‘mindless’ neuroscience and ‘brainless’ psychology are both incomplete explanatory frameworks (Holmes et al., 2014, Roiser, 2015). We also need to begin to connect the dots between the initiatives that appear to be rising and gaining momentum across areas and disciplines in mental health, One such example is the ROAMER roadmap for mental health research in Europe (Forsman et al., 2015, Haro et al., 2014).Another example is the proposal in March 2017 by Joshua Gordon, Director of the USA's NIMH (National Institute of Mental Health) to pay increased attention to psychosocial interventions and experimental therapeutics (Gordon, 2017).

### 7.5 Summary

In summary, psychological therapies are a learning process with a biological basis in the brain. Current approaches are not fully satisfactory. There is an imperative to understand why not. And when psychological therapies do work we need to understand why this is the case, and how we can improve them. There is a need to use scientific methods to achieve this. Meanwhile our patients are waiting. As Andrew Solomon expressed it:

*“I want to say that the treatments we have for depression are appalling. They're not very effective. They're extremely costly. They come with innumerable side effects. They're a disaster. But I am so grateful that I live now and not 50 years ago, when there would have been almost nothing to be done. I hope that 50 years hence, people will hear about my treatments and be appalled that anyone endured such primitive science.”*
